# A New Infrared Heat Treatment on Hot Forging 7075 Aluminum Alloy: Microstructure and Mechanical Properties

**DOI:** 10.3390/ma13051177

**Published:** 2020-03-06

**Authors:** Yi-Ling Chang, Fei-Yi Hung, Truan-Sheng Lui

**Affiliations:** Department of Materials Science and Engineering, National Cheng Kung University, Tainan 701, Taiwan; angelachangyiling@gmail.com (Y.-L.C.); luits@mail.ncku.edu.tw (T.-S.L.)

**Keywords:** hot forging 7075 aluminum alloy, rapid heating, infrared heating, long time high-temperature

## Abstract

When hot forging 7075 aluminum alloy, as a military material durable enough for most of its applications, it needs to be heat-treated to ensure the target material property achieves the application requirements. However, the material properties change because of heat throughout usage. In this study, a new approach was devised to heat treat the alloy to prevent material property changes. The study further clarified the effect of rapid heat treatment on the high-temperature resistance of a hot forging 7075 aluminum alloy. Infrared (IR) heat treatment was used as a rapid heating technique to effectively replace the conventional resistance heat (RH) treatment method. Our experimental result showed that IR heat treatment resulted in better age hardening at the initial aging stage, where its tensile strength and elongation appeared like that of a resistance heat treatment. More so, based on hardness and tensile test results, the IR-heated treatment process inhibited the phase transformation of precipitations at a higher temperature, improving high-temperature softening resistance and enhancing the thermal stability of the hot forging 7075 aluminum alloy.

## 1. Introduction

The 7075. (Al-Zn-Mg-Cu) aluminum alloy is extensively used in the aerospace industry and military because of its high strength and lightweight [1,2]. The 7075 aluminum alloy is a heat treatable aluminum alloy. Heat treatment can enhance its hardness and strength through the precipitation hardening. The precipitation hardening sequence of 7075 aluminum alloy is a supersaturated solid solution (S.S.S.S) → Guinier-Preston (GP) zones → metastable phase (η’) → equilibrium phase (η) [3,4,5]. The major strengthening phases are GP zones, η’ and η (MgZn_2_) phases. At the peak age (T6) condition, the major strengthening phases are GP zones and η’ phase, and at the T7x aging condition, the major strengthening phase is the η phase [4].

In this study, IR heat treatment was used as a rapid heating method. An IR heat treatment furnace not only can conserve energy but it can also provide a faster heating effect [6]. Previous studies on 6082 aluminum alloy showed that rapid heating could shorten the heat treatment duration and produce a high solute concentration, which leads to more uniform and fine precipitates in the matrix under the T6 condition [7,8,9]. These precipitates can improve strength and hardness [8]. However, how the IR heated effect on the hot forging 7075 aluminum alloy has not been investigated. Thus, in this study, IR heat treatment was used to try to upgrade the concentration of solutes and change the precipitates conditions of hot forging 7075 aluminum alloy. Since 7075 aluminum alloy is used in the military, tensile strength after the high-temperature treatment was also investigated.

We expected that IR heat treatment could delay the occurrence of high-temperature softening, effectively improving the high-temperature resistance of T6 materials. The results can be used as a reference in military and aerospace industry applications.

## 2. Experimental Procedures

**Figure 1:**
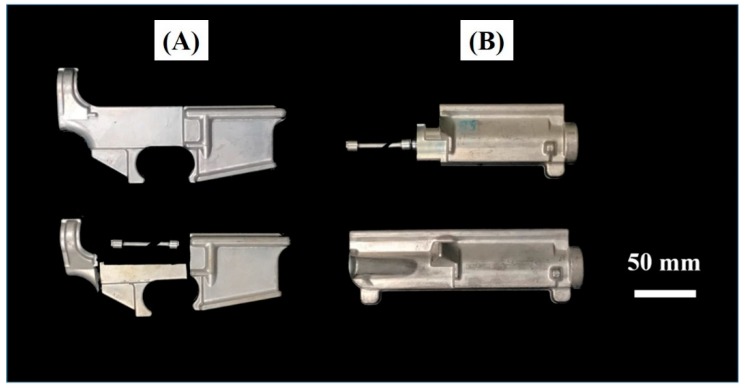
Photographs of 7075 aluminum hot forging parts.

We used commercial hot forging 7075 aluminum alloy. The hot forging 7075 forgings were made from 52 mm diameter cylindrical extruded rods, where the chemical composition is shown in Table 1. To make sure the experiment results can be used in different shapes of hot forging 7075 aluminum parts, two different shapes of parts were used in this study. Photographs of hot forging 7075 aluminum parts are shown in Figure 1.

**Figure 2:**
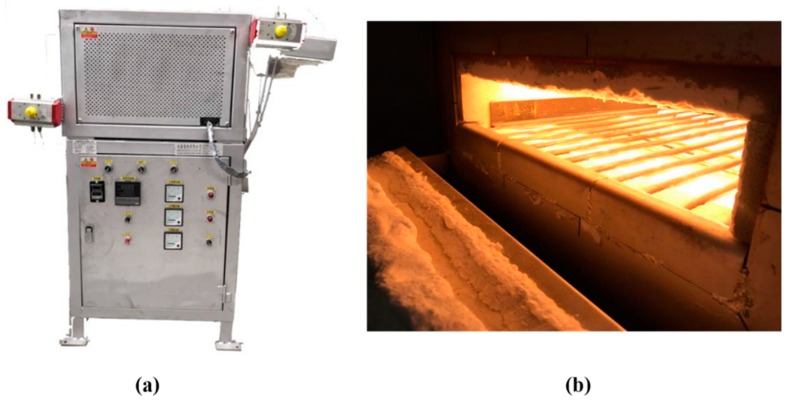
Photographs of infrared (IR) heat treatment apparatus: (**a**) IR heating apparatus, (**b**) heating space of the IR heating apparatus.

The heat treatment was performed in an infrared (IR) heating furnace and a resistance heating (RH) furnace (RH, CM30S, CHENG SANG, Changhua, Republic of China (Taiwan)), respectively. The IR heating furnace was developed at our laboratory. Figure 2 shows photographs of the IR heating furnace. The heating rate of the IR heating furnace has been published in our previous research [7].

Conventional artificial aging parameters [10,11] were utilized to establish baseline information (i.e., solution heat treatment conditions: RH/480 °C_60 min, aging conditions: RH/120 °C_24 h). The heat treatment conditions are shown in Table 2 and Table 3. To evaluate high-temperature softening, post heat treatment (200 °C for 240 min) was performed in the RH furnace after T6 heat treatment.

Metallographic investigations of the microstructural features and grain size were conducted using optical microscopy (OM, OLYMPUS BX41M-LED, OLYMPUS, Tokyo, Japan). All specimens (with the top surface perpendicular to the extrusion direction) were polished using SiC papers from #120 to #4000 grit in an Al_2_O_3_ aqueous suspension (1.0 and 0.3 μm) and a SiO_2_ polishing suspension and etched using Keller’s reagent.

The secondary phases of the alloy after heat treatment were observed using scanning electron microscopy (SEM, SU-5000, HITACHI, Tokyo, Japan), with energy-dispersive X-ray spectroscopy (EDS, EDAX, Singapore) and X-ray diffraction (XRD, Bruker AXS Gmbh, Karlsruhe, Germany). XRD analysis with Cu Kα radiation was employed in the 2θ range of 30°–55° to identify the secondary compounds.

Hardness measurements were performed on heat-treated samples using the Rockwell hardness (HR) test (Mitutoyo, Kawasaki-shi, Japan). The measurement conditions for the HR test followed the B-scale. The mean value of five impressions was taken as the hardness of the corresponding condition.

**Figure 3:**
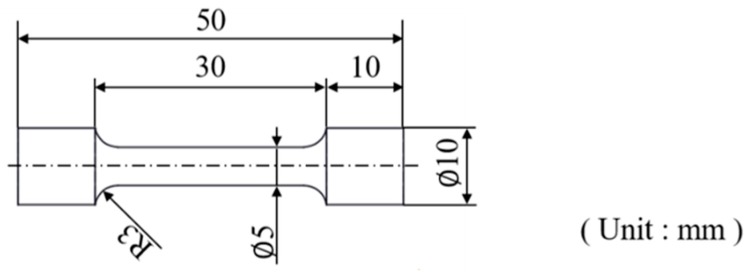
Diagram of the tensile test specimens.

All tensile tests were performed using a universal testing machine at room temperature with a stretching rate of 5 mm/min. The dimensions of the tensile test specimen are shown in Figure 3. Young’s modulus values were calculated from Young’s region of the tensile test graph, based on the formula as follows: E = ∆σ/∆ε.

## 3. Results and Discussion

### 3.1. Microstructure Observation

**Figure 4:**
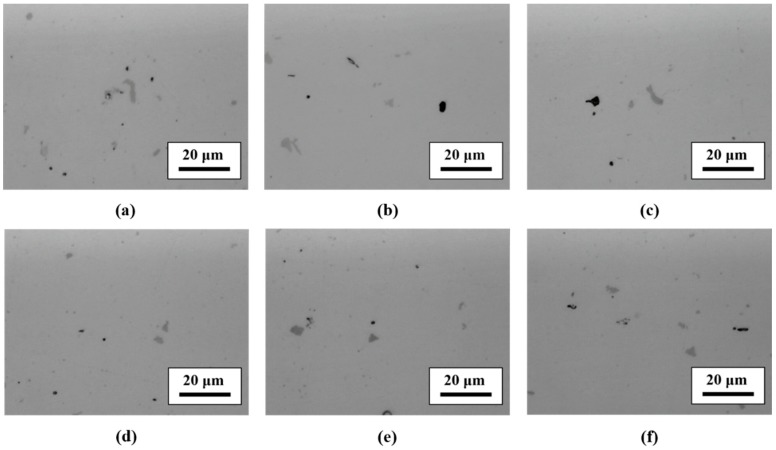
Microstructure observation results of specimens (forging A: (**a**–**c**), forging B: (**d**–**f**)). (**a**) As-received A, (**b**) RA120, (**c**) IA120, (**d**) as-received B, (**e**) RB120, and (**f**) IB120.

Figure 4a,d shows the microstructure observation results of A and B forgings. Two kinds of intermetallic compounds could be distinguished for all specimens (gray and black second phases particles) that could be observed. These second phase particles were distributed evenly in the matrix in both A and B forgings. There was no second phase distribution difference between the A and B forgings.

Figure 4b,c shows the microstructure observation results of heat-treated (480 °C, 120 min) A forging; RH-heated (Figure 4b) and IR-heated (Figure 4c), comparing RH-heated and IR-heated specimens, where there was no difference between them.

Figure 4e,f shows the microstructure observation results of heat-treated (480 °C, 120 min) B forging; RH-heated (Figure 4e) and IR-heated (Figure 4f), comparing RH-heated and IR-heated specimens, where there was no difference between them.

**Figure 5:**
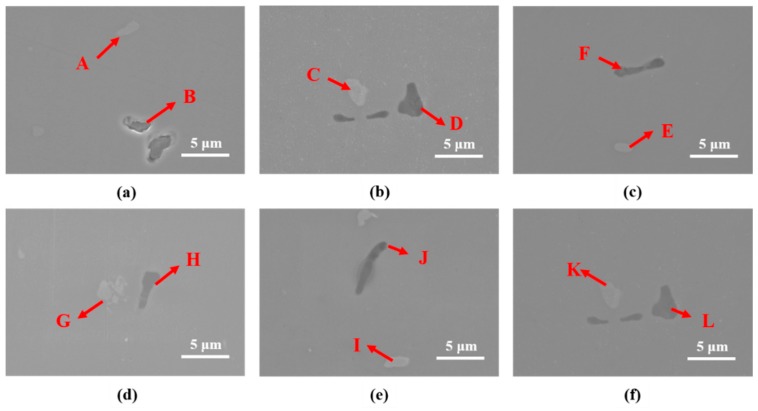
SEM observation results for various heat-treated specimens (forging A: (**a**–**c**), forging B: (**d**–**f**)). (**a**) As-received A, (**b**) RA120, (**c**) IA120, (**d**) as-received B, (**e**) RB120, and (**f**) IB120.

Figure 5 and Table 4 and Table 5 show the results of SEM observation and EDS analysis. According to the EDS analysis results, the gray compound (A, C, E, G, I and K compounds of Figure 5) was Al-Cu-Cr-Fe, and the black compound (B, D, F, H, J and L compounds of Figure 5) was Al-Mg-Si-Cr. According to previous research [12], Al-Cu-Cr-Fe and Al-Mg-Si-Cr compounds formed during casting and were rearranged during the hot forging process. Both Al-Cu-Cr-Fe and Al-Mg-Si compounds were stable at 480 °C. Thus, Al-Cu-Cr-Fe and Al-Mg-Si-Cr compounds remained after heat treatment at 480 °C for 120 min (Figure 4b,c,e,f).

**Figure 6:**
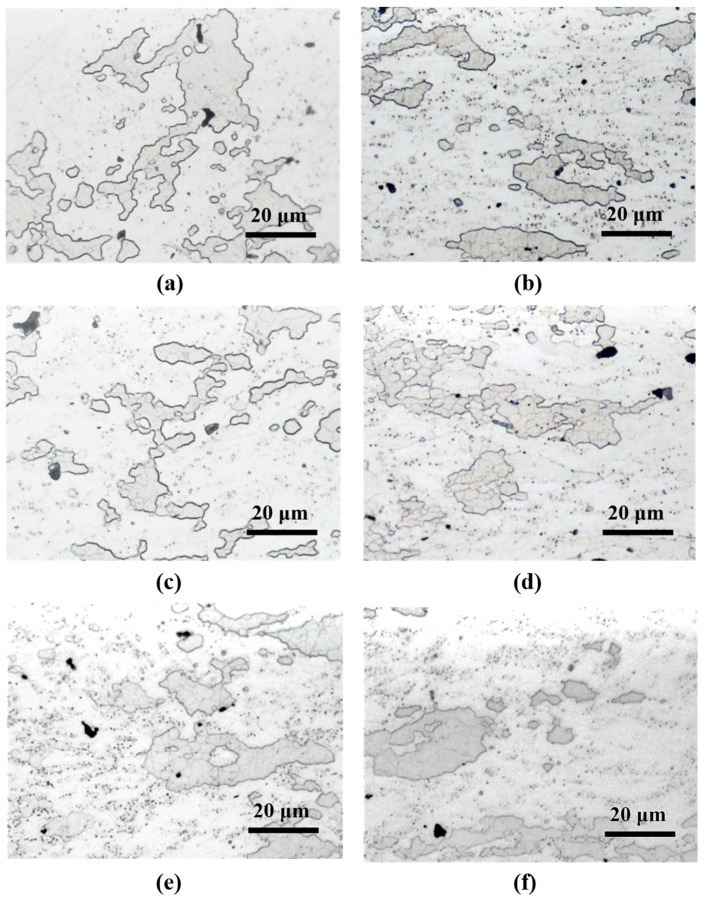
Metallographic images of various heat-treated specimens. (**a**) As-received A, (**b**) as-received B, (**c**) RA120, (**d**) RB120, (**e**) IA120, and (**f**) IB120.

Figure 6 shows the microstructure features of samples subjected to various heat treatments. Their microstructure features are similar; thus, hardness tests were performed.

### 3.2. Effect of High-Penetrating Infrared on Solution Heat Treatment

**Figure 7:**
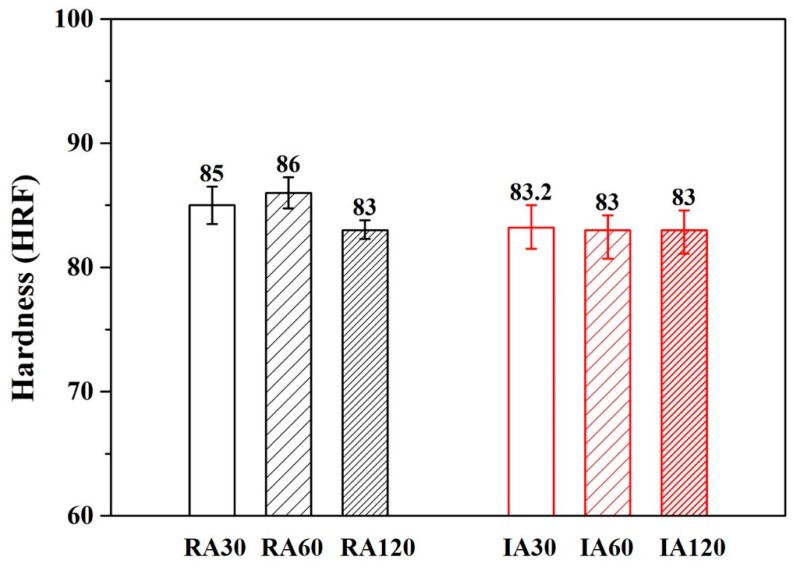
Hardness of various heat-treated forging A specimens.

**Figure 8:**
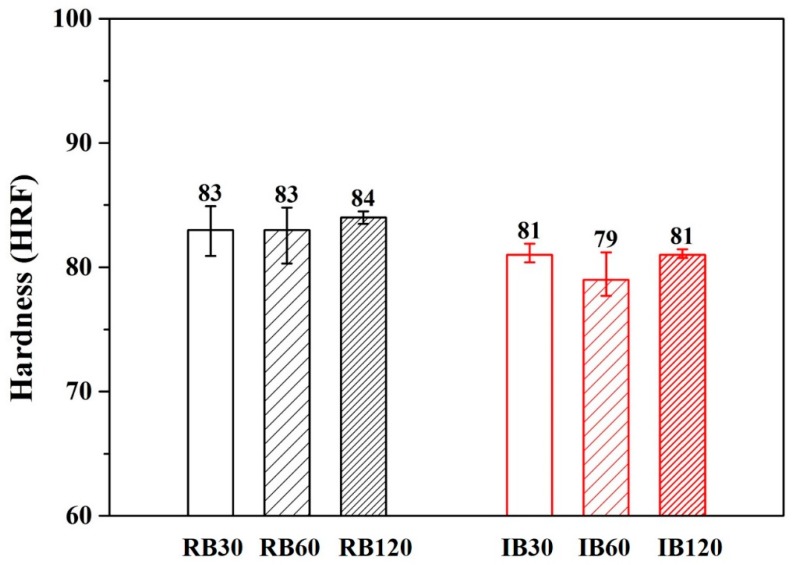
Hardness of various heat-treated forging B specimens.

Figure 7 and Figure 8 show the hardness results of forging A and forging B after various heat treatments. The hardness values of forging A specimens were higher than the values of forging B specimens. This result was due to the difference in metal flow during the forging process (different shapes are subjected to different stresses). It should be noted that for both RH- and IR-heated specimens, hardness after 30 min of treatment was the same as that after 120 min.

According to SEM (Figure 5) and EDS (Table 4 and Table 5) results, no coarse proeutectic MgZn_2_ phases formed in the material, indicating that proeutectic MgZn_2_ formed a smaller precipitated phase during the extrusion and forging process. Thus, only 30 min was needed for the solutionization.

**Figure 9:**
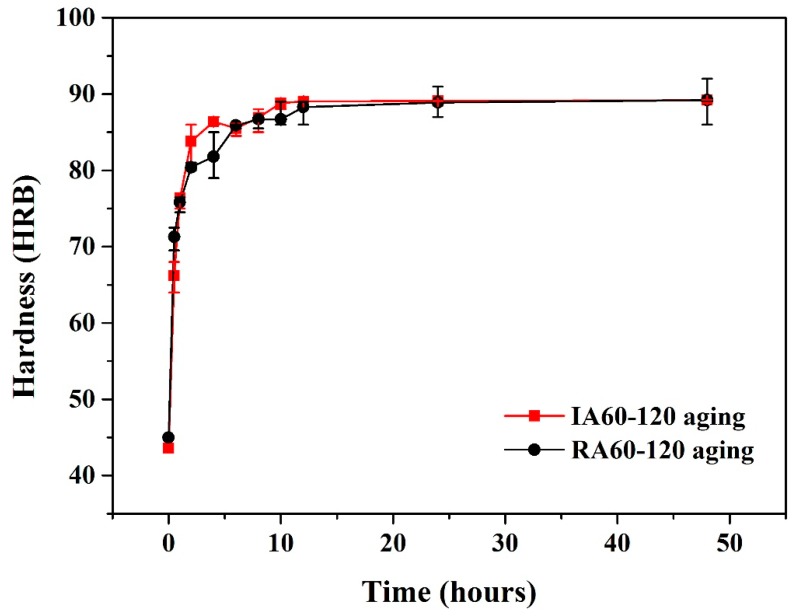
Effect of aging temperature and aging method on hardness profiles of specimens.

Figure 9 shows the hardness curves of IR- and RH-heated forging A specimens. A lot of research has been reported on the aging hardening of the 7075 aluminum alloy. The highest values of hardness developed by age hardening could be attributed to precipitation of coherent and finely dispersed precipitates (i.e., MgZn_2_) phases. These precipitates cause lattice distortions that make the alloy harder and act as obstacles to dislocation movement and thereby strengthen the alloy [10,13]. There was no difference in the peak age hardness value of IR- and RH-heated specimens, indicating that using the IR-heated method could provide the solution effect as RH-heated provide.

However, the IR-heated specimens showed a higher hardness value at the early stage of aging hardening compared to that obtained with RH. Similar results can be found in our previous research [8,9]. This phenomenon might be associated with a high concentration of solutes in the matrix.

**Figure 10:**
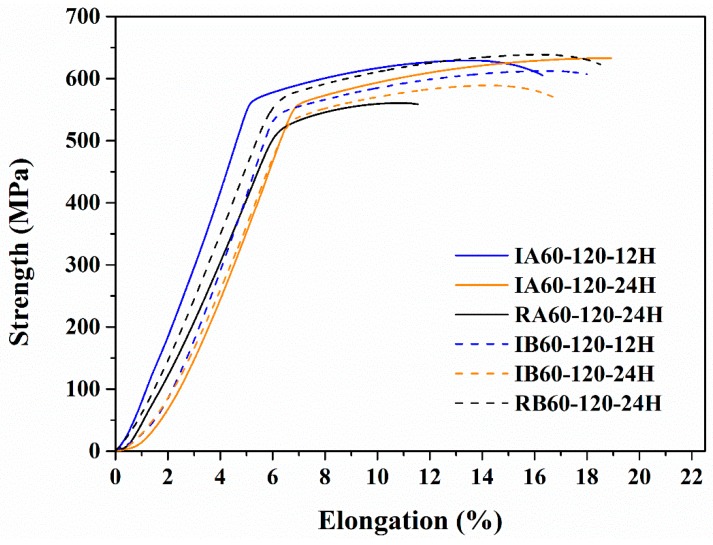
Tensile strength curves of various specimens.

Figure 10 shows the results of the tensile test. It could be observed that IR heat treatment gave the material tensile strength and ductility like that of the RH-heated material. It should be noted that the IR-heated specimens artificially aged for 12 h (IA60-120-12H/IB60-120-12H) and had strength and elongation similar to that of RH-heated specimens artificially aged for 24 h (RA60-120-24H/RB60-120-24H). Thus, IR heat treatment could shorten the artificial aging time to 12 h.

IR heat treatment enhanced Young’s modulus of the specimens (Table 6). As IR-heated and RH-heated specimens were of similar chemical composition, the difference of Young’s modulus was influenced by the other difference of the material, for example, the concentration of precipitates, the type of precipitates, and the related effect occurred by precipitates. Xie et al. [14] found that Young’s modulus of a material is related to the concentration of solutes and precipitates. Thus, according to the results of Table 6, we find that using IR-heated can result in the difference of precipitate. Increasing Young’s modulus can enhance the potential of thinning thickness of workpiece and vibration resistance [15]. Thus, IR heat treatment improves rolling and vibration resistance.

### 3.3. High-Temperature Resistance

**Figure 11:**
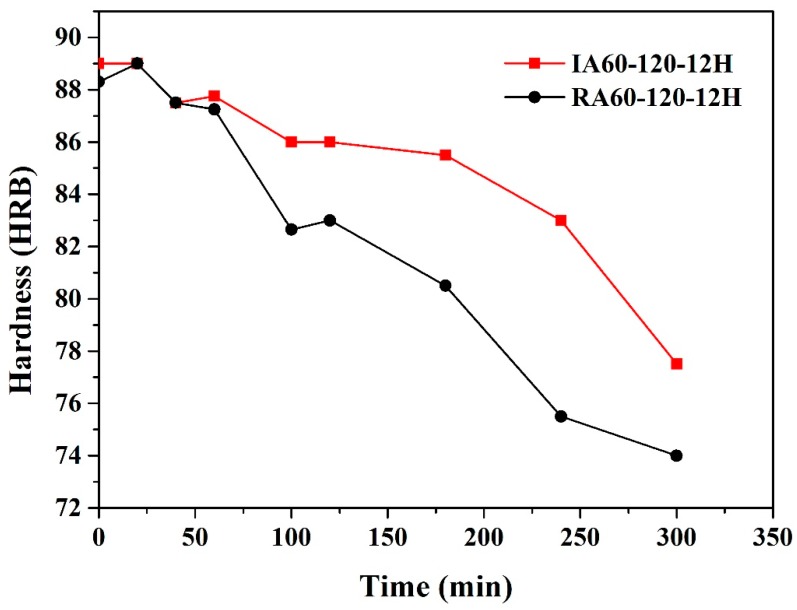
Effect of heating method on hardness profiles of specimens aged at 200 °C.

In this study, 200 °C was used as the high-temperature resistance test temperature. Karaaslan et al. [16] found that when the temperature reaches 200 °C, GP zones dissolve, and the material softens. However, the material is strengthened by the precipitation of the η’ phase in the following stage. Strength is enhanced by η’ precipitates, and the material softens when the η’ precipitates become the η phase. The material also softens when over-aging occurs [5]. Danh et al. [5] found that at 200 °C, GP zones dissolve within 10 min and over-aging occurs after 30 min. Figure 11 shows the time and hardness profiles of 200 °C heating of T6 (IA60-120-12H and RA60-120-12H) specimens. The hardness increases within 30 min due to the re-precipitation and phase transformation of GP zones. Over-aging also occurs within 30 min. It should be noted that the IR-heat-treated specimen has a slower softening rate at high temperatures.

**Figure 12:**
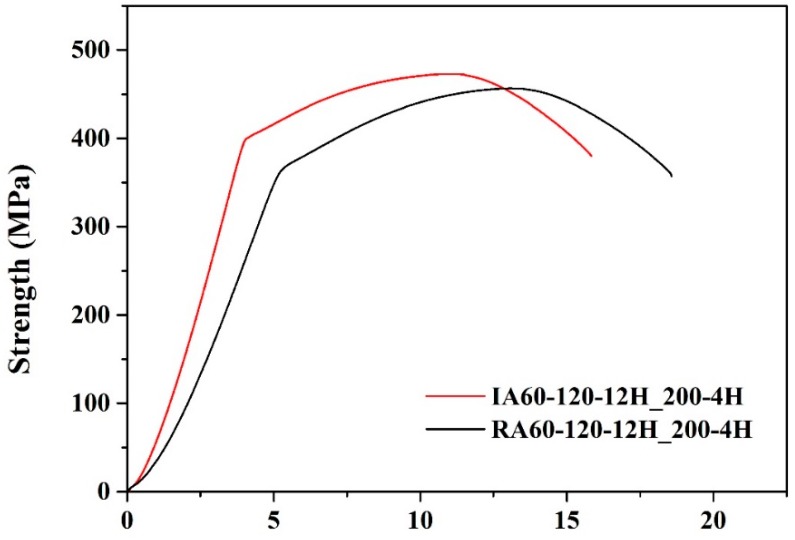
Strain-stress curves of specimens after the high-temperature test (200 °C, 4 h).

Figure 12 and Table 7 show the results of the tensile test of high-temperature resistance test specimens. The tensile test was carried out after heat treatment at 200 °C for 4 h. We found that the tensile strength decreased, and ductility increased, indicating that over-aging occurred for both specimens. Additionally, IA60-120-12H_200-4H showed higher strength (yield strength, YS: 400 MPa, ultimate tensile strength, UTS: 474 MPa) than that of RA60-120-1F2H_200-4H (YS: 367 MPa, UTS: 458 MPa), indicating better high-temperature resistance. Even after heat treatment at 200 °C for 240 min, the IR-heated specimen showed a better Young’s modulus than that of the RH-heated specimen.

**Figure 13:**
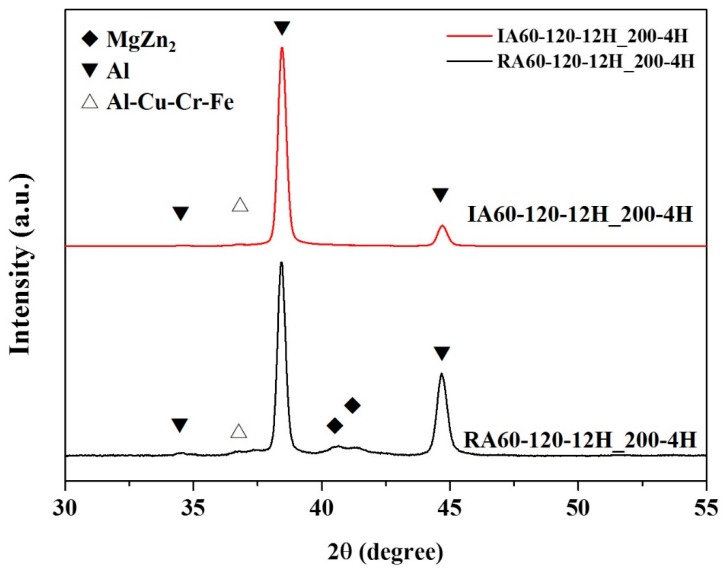
XRD patterns of specimens after the high-temperature test (200 °C, 4 h).

In the XRD patterns (shown in Figure 13), according to our previous research [11], MgZn_2_ and Al-Cu(Cr)Fe compounds could be observed. Comparing RA60-120-12H_200-4H and IA60-120-12H_200-4H specimens, RA60-120-12H_200-4H has a more obvious MgZn_2_ peak compared to that for IA60-120-12H_200-4H. By comparing to the results in Figure 11, there are more MgZn_2_ formed in the RA60-120-12H_200-4H; while MgZn_2_ is representative of over-aging.

This study investigated over-aging with RH and IR heat treatments at 200 °C. The IR-heated specimen showed better high-temperature resistance. According to previous investigations [7], IR-heated specimens had relatively uniform and dense precipitations during the precipitate process.

Thus, it is reasonable to infer that the ability to withstand high temperature is due to the uniform and dense precipitations which formed due to the IR heated process. That is, these fine and larger amount of precipitations (compared to the RH heated sample) consumed the Mg and Zn atoms in the matrix and delayed the transformation to η-MgZn_2_.

Moreover, from the results of mechanical tests as presented in Figure 9 and Figure 10; higher hardness values at the initial aging stage, the enhancement of tensile strength, and Young’s modulus, our experiment results chime in with the results using IR as a heated method that can bring about a fine and larger amount of precipitations.

## 4. Conclusions

In this study, a systemic study on infrared heat treatment on hot forging 7075 aluminum alloy, the following findings were obtained:

1. IR heat treatment can be used as a heat treatment method on hot forging 7075 aluminum alloy. Moreover, compared to the RH-heated method, the IR-heated method results in better age hardening at the initial aging stage.
2. IR heat treatment shortens the heat treatment time and produces strength and elongation in 12 h, like that obtained with RH in 24 h.
3. IR-heated specimens had a higher Young’s modulus than that of RH-heated specimens. IR-heated can change the precipitates’ condition of the specimens.
4. IR-heated specimens had better high temperature softening resistance than RA-heated specimens.

## Figures and Tables

**Table 1:** Chemical composition (wt.%) of 7075 aluminum alloy.

Element	Al	Fe	Si	Cu	Mg	Mn	Zn	Cr	Ti
wt.%	Bal.	0.15	0.08	1.26	2.21	0.03	5.39	0.2	0.05

**Table 2:** Experimental parameters and specimen codes (only solution heat treatment).

Experiment Condition	Heating Method	SHT	Code
A (grip)	RH	480 °C, 30, 60, 120 min	RA30, RA60, RA120
	IR	480 °C, 30, 60, 120 min	IA30, IA60, IA120
B (upper receiver)	RH	480 °C, 30, 60, 120 min	RB30, RB60, RB120
	IR	480 °C, 30, 60, 120 min	IB30, IB60, IB120

(SHT: solution heat treatment; RH: resistance heating; IR: infrared)

**Table 3:** Experimental parameters and specimen codes.

Experiment Condition	Heating Method	SHT	Artificial Aging	Code
A (grip)	RH	480 °C, 60 min	120 °C, 0–24 h	RA60-120_XH
		480 °C, 60 min	140 °C, 0–24 h	RA60-140_XH
	IR	480 °C, 60 min	120 °C, 0–24 h	IA60-120_XH
		480 °C, 60 min	140 °C, 0–24 h	IA60-140_XH
B (upper receiver)	RH	480 °C, 60 min	120 °C, 0–24 h	RB60-120_XH
		480 °C, 60 min	140 °C, 0–24 h	RB60-140_XH
	IR	480 °C, 60 min	120 °C, 0–24 h	IB60-120_XH
		480 °C, 60 min	140 °C, 0–24 h	IB60-140_XH

X: heating duration (hours); SHT: solution heat treatment; RH: resistance heating; IR: infrared.

**Table 4:** EDS analysis results for specimens as-received A, RA120, and IA120, as shown in Figure 5a,c,e, respectively.

at.%	As-received A		RA120		IA120	
	A	B	C	D	E	F
Fe	7.8	0.0	8.3	0.1	0.2	11.5
Cu	3.1	0.1	3.1	0.2	0.2	3.1
Zn	1.2	1.1	1.4	1.7	0.5	1.8
Mg	1.3	5.1	1.0	19.1	42.6	1.5
Al	80.6	50.7	82.3	46.6	32.5	81.4
Si	2.8	40.4	0.5	30.4	23.8	0.5
Cr	3.2	2.6	3.4	1.9	0.2	0.2

**Table 5:** EDS analysis results for specimens as-received B, RB120, and IB120, as shown in Figure 5d–f, respectively.

at.%	As-received B		RB120		IB120	
	G	H	I	J	K	L
Fe	8.0	0.2	8.5	0.0	12.2	0.2
Cu	2.6	0.4	3.8	0.2	3.4	0.2
Zn	1.7	1.0	1.0	0.9	1.6	0.5
Mg	0.8	33.3	1.2	40.8	1.5	42.6
Al	82.8	41.6	82.6	36.0	80.6	32.5
Si	0.4	22.1	0.7	20.6	0.4	23.8
Cr	3.7	1.4	2.2	1.5	0.3	0.2

**Table 6:** Specific tensile strength, elongation, and hardness from Figure 10.

Material Properties	YS (MPa)	UTS (MPa)	UE (%)	TE (%)	Young’s Modulus (σ/ε, MPa)	Hardness (HRB)
Sample						
IA60-120-12H	574	630	7	10	121	89
IA60-120-24H	507	570	7	10	108	88
RA60-120-24H	512	554	4	5	100	89
IB60-120-12H	528	613	9	10	122	90
IB60-120-24H	533	634	7	10	161	88
RB60-120-24H	554	638	9	12	107	89

**Table 7:** Specific tensile strength, elongation, and hardness of high-temperature (200 °C) over-aged specimens.

Material Properties	YS (MPa)	UTS (MPa)	UE (%)	TE (%)	Young’s Modulus (σ/ε, MPa)	Hardness (HRB)
Sample						
IA60-120-12H_200-4H	400	474	7	12	124	76
RA60-120-12H_200-4H	367	458	7	13	89	83
